# Path Diagnostic Therapeutic Care (PDTA) in children and adolescents with headache

**DOI:** 10.1186/1824-7288-40-S1-A85

**Published:** 2014-08-11

**Authors:** Vincenzo Raieli, Angelo Vecchio, Flavia Consolo, Giuseppe Santangelo, Renata Pitino, Giuseppe Porrello, Francesca Vanadia

**Affiliations:** 1 Department of Childhood and Adolescent Neuropsychiatry of ASP6, Palermo, 90100, Italy; 2 Department of Childhood and Adolescent Neuropsychiatry Di Cristina ARNAS Civico, Palermo, 90100, Italy

## Background

Headache is a common and very disabling disease in pediatric population and also its management in ambulatory and emergency pediatric unit has a significant economic and social impact.

For these reasons, it seemed appropriate to identify care pathways involving different specialists of hospital and local healthcare services that collaborate together to improve diagnosis and management of headache in young people. “Path Diagnostic Therapeutic Care (PDTA) in children and adolescent with headache” is a (appropriate and individualized) care pathway involving a network of different medical structures specialized for neuropsychiatric disease in childhood.

## Methods

The purpose of the PDTA in children and adolescents with headache is to reinforce collaboration between hospital and territorial neuropsychiatric services, through the creation of dedicated and appropriate paths; to encourage patients to take a proactive approach to the management of their headache in to the territorial healthcare service, already in the hospital, implementing shared diagnostic and therapeutic project. PDTA targets are patients with acute headache (primary or secondary), which require hospitalization at the Department of pediatric neuropsychiatry (NPI) ARNAS “Di Cristina” Palermo and patients which require NPI territorial healthcare services, through shared diagnostic and therapeutic pathways.

Established paths:

1) Inside the territorial services: path from level I territorial surgery to Level II specialized department of "Diagnosis and treatment of headaches in children and adolescents";

2) From territorial services to hospital: path from level II specialized department “Diagnosis and treatment of headaches in children and adolescents" to Department of pediatric neuropsychiatry (NPI) ARNAS “Di Cristina” Palermo;

3) From hospital to territorial services: path from Department of pediatric neuropsychiatry (NPI) ARNAS “Di Cristina” Palermo to “Level II specialized territorial department of Diagnosis and treatment of headaches in children and adolescents";

4) Inside the territorial services: path from "Level II specialized department: Diagnosis and treatment of headaches in children and adolescents " to “level I NPIA territorial surgery“

## Results

According to data collected, PDTA implementation will permit to reduce by 20% of inappropriate accesses to Emergency Pediatric Unit (roughly 50% admitted primary headaches) and by 15% reduction of neuroradiological examinations performed in the emergency department.

## Conclusions

PDTA propose to improve sanitary assistance in children and adolescent with headache and his parents, through a appropriated and dedicated diagnostic and therapeutic paths. Furthermore, the main expected result is improving patient compliance to diagnosis and treatment of headache in pediatric population

